# Royal Jelly Delays Motor Functional Impairment During Aging in Genetically Heterogeneous Male Mice

**DOI:** 10.3390/nu10091191

**Published:** 2018-09-01

**Authors:** Nobuaki Okumura, Toshihiko Toda, Yusuke Ozawa, Kenji Watanabe, Tomoki Ikuta, Tomoki Tatefuji, Ken Hashimoto, Takahiko Shimizu

**Affiliations:** 1 Institute for Bee Products and Health Science, Yamada Bee Company, Inc., Okayama 708-0393, Japan; no1780@yamada-bee.com (N.O.); ti1340@yamada-bee.com (T.I.); tt0880@yamada-bee.com (T.T.); kh0663@yamada-bee.com (K.H.); 2 Department of Clinical Cell Biology and Medicine, Chiba University Graduate School of Medicine, Chiba 260-8670, Japan; hik_toda@proteome.jp (T.T.); ozawayusuke3@gmail.com (Y.O.); kng.wtnb@chiba-u.jp (K.W.)

**Keywords:** royal jelly, aging, motor function, muscle stemness, muscle atrophy, genetically heterogeneous mice

## Abstract

Aging is associated with motor disorders that decrease the quality of life (QOL). Royal jelly (RJ), used as a dietary supplement, has shown various health benefits and, therefore, it has the potential to improve the QOL during aging. We have previously developed protease enzyme-treated RJ to avoid the anaphylactic response induced by RJ supplementation. However, the effects of a lifelong treatment with RJ on normal aging have not been fully clarified. In this study, we investigated the effects of enzyme-untreated RJ (NRJ) and enzyme-treated RJ (ERJ) on the aging process focusing on motor functions, by using a genetically heterogeneous (HET) mouse model experimentally endowed with genetic diversity. We performed four different physical performance tests (grip strength, wire hang, horizontal bar, and rotarod). We showed that the age-related impairment of the motor functions was significantly delayed in RJ-treated mice. Both NRJ and ERJ were similarly effective against these types of aging-associated declines. Histological analyses revealed that the RJ treatment affected the muscle fiber size at an advanced age. We also demonstrated that age-related changes in muscle satellite cell markers and catabolic genes were affected in RJ-treated mice. These results suggest that non-protein components of RJ improved the motor function in aging mice. These findings indicate that RJ has the potential to change the QOL during aging by regulating the motor function.

## 1. Introduction

In aging societies worldwide, the number of elderly people suffering from age-associated diseases, such as motor disorders, metabolic syndrome, and cognitive decline, is increasing [1,2,3,4]. These diseases are directly associated with a reduced quality of life (QOL), which generates a distinction between the lifespan and a healthy lifespan. To overcome these issues, dietary interventions, such as dietary restriction, drugs, and nutrients, are expected to slow the rate of aging and lead to healthy aging [5,6]. 

In honey bee (*Apis mellifera*), royal jelly (RJ) determines epigenetic changes leading to different fates in worker bees and long-lived queen bees with identical genotypes [7,8,9,10]. RJ is produced by the hypopharyngeal and mandibular glands of nursing worker bees. RJ might be a nutraceutical food with prolongevity activity, because the supplementation of honey bee larvae with RJ induces development into a queen bee, which has a characteristic larger body and a longer life than the workers [11]. In addition, RJ and its ingredients have been identified as life-extending factors by other studies using invertebrates [12,13,14,15]. RJ also reportedly prolongs the lifespan of C3H/HeJ mice [16]. RJ is expected to moderate the healthy lifespan in humans, as it has been reported to have a variety of health benefits, such as longevity promotion, anti-depression, anti-dry eye, anti-obesity, and anti-sarcopenia activities in animal models as well as in humans [16,17,18,19,20,21]. Therefore, RJ is a candidate substance for extending the healthy life expectancy. However, the effects of a lifelong treatment with RJ on the healthy lifespan during normal aging have not been fully elucidated.

It is reported that some individuals undergo an anaphylactic response following RJ supplementation. To overcome this issue, we have previously developed enzyme-treated RJ (ERJ). By treating RJ with alkaline proteases, the allergic reaction to RJ was reduced without nutritional loss of minerals, vitamins, and RJ-specific fatty acids like 10-hydroxy-2-decenoic acid (10-HDA) [22]. However, it has not been clarified whether the health benefits of RJ are maintained after protease treatment.

To identify the compounds that delay aging, experimental animal models have been employed. Numerous studies have reported findings of an extended lifespan in mice [23]. However, those studies were conducted on mice with a uniform genetic background; we therefore needed to carefully consider whether or not the effects on lifespan were genotype-dependent. In recent years, a genetically heterogeneous (HET) mouse model has been used to examine agents that are expected to increase the lifespan in intervention testing programs (ITPs) conducted by the National Institute of Aging (NIA) [24]. The HET model has several advantages over other models in aging research. Because HET mice are generated from four different inbred strains (BALBc, C57BL/6, C3H, and DBA/2), they have genetic diversity and exhibit various causes of death, similar to human populations. In ITPs, more than 10 compounds have been tested using the HET mouse model [25], with only rapamycin being found to increase longevity in both male and female animals [26]. Food ingredients, such as resveratrol, green tea extract, curcumin, medium-chain triglycerides, and fish oil, all failed to extend the lifespan or healthy lifespan [27,28]. 

In the present study, we investigated the effects of RJ on lifespan and healthy lifespan using HET mice. We compared the longevity effects of normal royal jelly (NRJ) and ERJ. To clarify the effects of a lifelong treatment with RJ, we started the treatments when the mice were six months old. We carried out various physical performance tests in mice to assess the ability of RJ to extend the animals’ healthy lifespan.

## 2. Materials and Methods 

### 2.1. Mouse Production and Maintenance

All the animal experiments performed in this study were approved by the ethics committee of Chiba University based on their internal guidelines. Genetically heterogeneous four-way cross mice were produced at Chiba University as described previously [24]. The background mouse strains, BALB/cCrScl, C57BL/6NCrSlc, and CB6F1/Slc (F1 hybrid between female BALB/cCrScl and male C57BL/6NCrSlc), were obtained from Japan SLC Inc. (Shizuoka, Japan). We first mated male DBA/2CrSlc with female C3H/HeSlc to obtain F1 hybrid C3D2F1/CU. We then mated male C3D2F1/CU with female CB6F1/Slc to produce genetically heterogeneous mice (Supplementary Figure S1). Mice were weaned at 4 weeks of age and then housed in a plastic cage at 5–6 mice/cage under a 12 h light–dark cycle at room temperature. Mice were given free access to water with a plastic bottle and fed an MF pellet diet (Oriental Yeast Co., Ltd., Tokyo, Japan) ad libitum. 

### 2.2. Experimental Diet and RJ Treatment

Lyophilized raw RJ (enzyme-untreated RJ (NRJ, Lot No. YRP-M-140830) and enzyme-treated RJ (ERJ, Lot No. YDP-M-140423)) were prepared at Yamada Bee Company, Inc. (Okayama, Japan). RJ was standardized with the amounts of specific fatty acids (E)-10-hydroxy-2-decenoic acid (10H2DA) and 10-hydroxydecanoic acid (10HDA): NRJ contained a minimum 3.8% of 10H2DA and a minimum 0.6% of 10HDA, while ERJ contained a minimum 3.5% of 10H2DA and a minimum 0.6% of 10HDA. The experimental diets were prepared by thoroughly mixing lyophilized RJ with MF powder diet (Oriental Yeast) at a concentration of either 0.05% (low dose) or 0.5% (high dose) (*v*/*v*). The general composition of the experimental diets is shown in Supplementary Table S1. The compositions of each diet were calculated from manufacturing information of MF powder diet, NRJ, and ERJ. 

We used male heterogeneous mice in this study. At 6 months of age, the mice were randomly separated into five groups, namely, Control (*n* = 66), 0.05% NRJ (NRJL, *n* = 62), 0.5% NRJ (NRJH, *n* = 66), 0.05% ERJ (ERJL, *n* = 64), and 0.5% ERJ (ERJH, *n* = 66), and were given the experimental diet ad libitum at this time. The diet was refreshed twice a week. 

### 2.3. Physical Performance Tests

**Figure 1:**
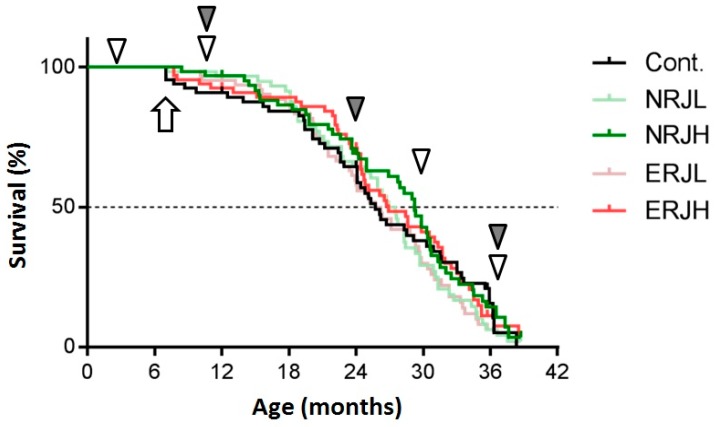
A comparison of the survival curves between control and royal jelly (RJ)-treated mice. A Kaplan–Meier analysis was used to estimate the median lifespan. The arrow indicates the beginning of the RJ treatment. The white arrowheads indicate the time at which mice physical performance was tested. The gray arrowheads indicate the time at which the mice were sacrificed to obtain samples for the experiments. Cont: control; NRJL: enzyme-untreated RJ low-dose; NRJH: enzyme-untreated RJ high-dose; ERJL: enzyme-treated RJ low-dose; ERJH: enzyme-treated RJ low high-dose.

We assessed the age-dependent physical performance of HET mice at 3, 9–10, 30–33, and 36–39 months of age (show Figure 1, white arrowheads). The muscle strength was tested with the forelimb grip strength and wire hang tests. In the grip strength test, each mouse grasped a grip-strength meter (spring coil) with their forelimbs and then gradually pulled backward until they released their grip. The maximum force was recorded in Newtons (N). The tests were repeated five times. The average force was determined as the individual mouse grip strength. In the wire hang test, the mouse was placed onto a wire mesh cage top, which was then gently inverted to encourage the mouse to grip the wire. The retention time was recorded. We set the cut-off at 120 s. Motor coordination was tested with a rotarod and a horizontal bar. In the rotarod test, the mice were adapted to a rotarod (Muromachi Kikai Co., Ltd., Tokyo, Japan) at 4 rpm for 1 min, after which the rotarod was lineally accelerated from 4 to 40 rpm in 5 min. The retention time was recorded. In the horizontal bar test, the tail of the testing mouse was held, and the mouse was placed onto the center of a steel bar (2.5 mm diameter) by its forelimbs, with the tail then gently released. The retention time was recorded. We set the cut-off at 60 s. The experiment was performed twice. 

### 2.4. Blood and Tissue Collection

Whole blood was collected at 10 and 24 months of age after 18 h fasting under anesthesia (combination of pentobarbital and ether). The number of blood cells was immediately measured after blood collection with an automatic hematology analyzer (Celltac MEK-4150; Nihon Kohden, Tokyo, Japan). EDTA-plasma was also prepared at the same time points to analyze plasma biochemical parameters: plasma albumin, aspartate aminotransferase (AST), alanine aminotransferase (ALT), total cholesterol (T-CHO) and triglyceride (TG). Tissues were removed from mice at 9–10, 24, and 36–39 months of age (show Figure 1, gray arrowheads). All tissues were perfused with ice-cold phosphate buffered saline (PBS) before removal and then flushed with liquid nitrogen. All samples were stored at −80 °C until analysis.

### 2.6. Histological Analyses

The skeletal muscles (gastrocnemius muscle) were fixed with paraformaldehyde, and cross sections were made. To evaluate the general muscle histology, the cross sections were stained with hematoxylin and eosin. Photographs were taken with the All-In-One fluorescent microscopic system, BZ-700 (Keyence, Osaka, Japan). The cross-sectional area (CSA) of muscle fibers and the number of centronuclei was analyzed with a BZ-Z analyzer (Keyence). 

### 2.7. Measurement of Bone Mineral Density

Bone mineral density (BMD) was measured at 9–10, 24, and 36–39 months of age by using a dual-energy X-ray absorptiometry method. Briefly, the mice were anesthetized with pentobarbital, and then the BMD of their femurs was measured using the PIXImus instrument (Lunar Corp., Madison, WI, USA).

### 2.8. Analyses of mRNA Expression

Total RNA was extracted from the gastrocnemius muscle at 10 and 24 months of age with TRIzol® RNA Isolation Reagents (Thermo Fisher Scientific K.K., Kanagawa, Japan). Total RNA was treated with RQ-1 RNase free-DNase (Promega, Madison, WI, USA) to remove the contaminated genomic DNA. The concentration of total RNA was quantified by NanoDrop (Thermo Fisher Scientific K.K). One microgram of total RNA was reverse transcribed with ReverTra Ace® qPCR RT Master Mix (Toyobo, Osaka, Japan). Real-time polymerase chain reaction (PCR) was performed by the fluorescent dye SYBR Green method using SsoAdvanced™ Universal SYBR® Green Supermix (Bio-Rad Laboratories, Inc., Hercules, CA, USA) with a Miniopticon real-time PCR system (Bio-Rad Laboratories). The primer pairs used in this experiment are shown in Supplementary Table S4. We evaluated the stability of five reference gene candidates using the “NormFinder” algorithm [29]: B2m, Actb, H2a, Gurb, and Rsp18. We selected H2a as a housekeeping gene because it showed the most stable expression in the skeletal muscle.

### 2.9. Statistical Analyses

All statistical analyses were performed with GraphPad Prism7. The data were represented as mean ± standard error of mean (SEM). The statistical differences among groups were analyzed by the non-parametric Kruskal–Wallis test (post-hoc Dunn’s multiple comparisons test). If two groups were analyzed, the significance was determined by the Mann–Whitney’s *U* test. The survival curves were calculated by the Kaplan–Meier method, and the statistical difference was determined by the log-rank test. The statistical analysis of each experiment is described into the figure legends.

## 3. Results

### 3.1. Food Intake, Body Weight, and Survival

The general compositions and total calories of the experimental diets were comparable among the groups (Supplementary Table S1). Food consumption gradually decreased with age in all groups, but it was similar among groups of the same age (Supplementary Figure S2). We calculated the RJ intake from the food consumption values. Mice consumed RJ at approximately 50 mg/kg body weight/day in the low-RJ group and 500 mg/kg body weight/day in the high-RJ group during the experiments (Supplementary Figure S2a, right panel). We measured the body weight every month from six months of age (beginning of RJ treatment). The body weight peaked around 15 months of age (9 months after RJ treatment) and then gradually decreased during the process of aging. The body weight in the ERJL group was significantly lower from 14 to 22 months of age than in the control group, but we did not explore the reasons for this weight reduction. There were no significant changes in the body weight in the other groups (Supplementary Figure S2b). We then estimated the effect of RJ on survival (Figure 1). High-dose RJ (both NRJH and ERJH) did not affect the median lifespan. Low-dose RJ showed no marked effect on survival. We did not notice any safety concerns related to the treatment with RJ in our evaluation of the plasma biochemical parameters and blood cells (Supplementary Tables S2 and S3).

### 3.2. Effects of RJ on the Age-Dependent Impairment of Physical Performance 

**Figure 2:**
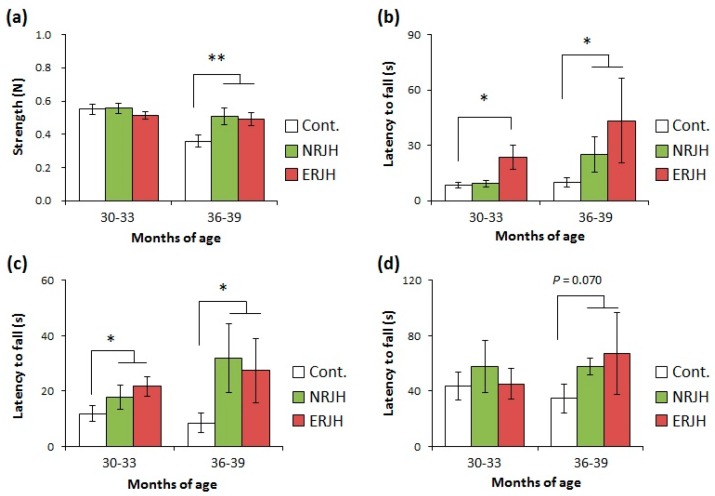
Royal jelly improved the age-related impairment of the motor function in genetically heterogeneous mice. The grip strength was measured by a spring gauge test (a) and a wire hang test (**b**). The locomotor activity was measured by a horizontal bar test (**c**) and an accelerated rotarod test (**d**). Data are shown as the mean ± standard error of mean (SEM) (*n* = 14–18 at 30–33 months or *n* = 4–7 at 36–39 months). ** *P* < 0.01 and * *P* < 0.05 vs. 9–10 months

In daily inspections, mice activity, such as rearing and hanging on the cage top, was higher for mice fed high-dose RJ than for control mice, even for relatively old animals (more than 24 months of age). We further analyzed the effects of RJ on the QOL by assessing the motor functions with different types of tests. Muscle strength decreased in an age-dependent manner in HET mice (Supplementary Figure S3). We also found that motor coordination decreased with age (Supplementary Figure S3). In particular, a significant impairment of the motor functions was observed in mice at 36–39 months of age in all tests. These results indicated that the rate of physical performance impairment with aging was slower than the rate of the lifespan. We next evaluated the effects of RJ in the age-dependent impairment of the motor functions at 30–33 and 36–39 months of age (Figure 2 and Supplementary Figure S4). We showed that the declines in both muscle strength and motor coordination were delayed in mice fed the high dose of RJ compared with control mice. There were no significant differences between the NRJ and ERJ groups, suggesting that the components that helped improve the QOL were not diminished by treating RJ with proteases. To understand the role of RJ in motor function impairment with age and obtain statistical power, we statistically compared the control group and the RJ (combined NRJ and ERJ)-treated group. RJ was found to significantly improve mice motor functions at 36–39 months of age (Figure 2).

### 3.3. Effects of RJ on the Age-Related Morphological Changes in Muscle Fiber Quality 

**Figure 3:**
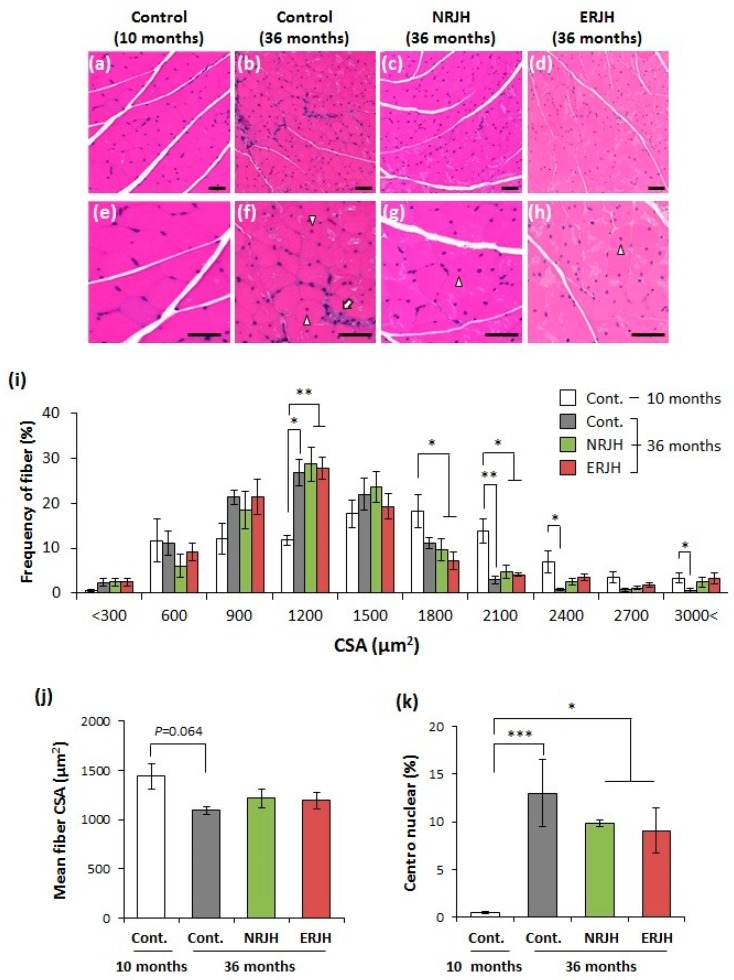
Effects of RJ on the age-related morphological changes in muscle fibers in genetically heterogeneous mice. A–D, Morphology of gastrocnemius muscle fibers (Hematoxylin–eosin (H & E) staining) of young (**a**,**e**), aged (**b**,**f**), aged NRJ-treated (**c**,**g**), and aged ERJ-treated (**d**,**h**) mice. The upper panels show a low magnification (**a**–**d**), and the lower panels show a high magnification (**e**,**f**). Fibers with centronuclei were observed in aged mice (see arrowheads in the figure). Inflammatory cell infiltration is indicated by the white arrow. The bar indicates 100 µm. (**i**), Distribution of the cross-sectional areas (CSA) of gastrocnemius muscle fibers. ** *P* < 0.01, * *P* < 0.05. (**j**), The mean fiber CSA was calculated from the average CSA of over 170 fibers in individual mice. Data are shown as the mean ± SEM (*n* = 5–8). (**k**), percentage of the muscle fiber with centronuclei. Data are shown as the mean ± SEM (*n* = 5–8). *P* values were determined by the Kruskal–Wallis test (post-hoc Dunn’s multiple comparisons test). *** *P* < 0.001, ** *P* < 0.01, * *P* < 0.05

We then investigated the mechanism underlying the effect of RJ on the delayed loss of motor function with age, focusing on the muscle function. The muscle weight decreased in an age-dependent manner in HET mice. The weight was significantly decreased (by 40%) at 36–39 months of age in accordance with motor functional loss (Supplementary Figure S5, upper-left pannel). However, RJ supplementation had no effect on the muscle weight loss (Supplementary Figure S5, upper-right panel). Similar results were obtained with regard to the bone mineral density (Supplementary Figure S5, lower pannels). We then performed a histological analysis of the muscles. Hematoxylin–eosin staining of aged muscle tissue sections from the control mice showed atrophic features, such as a reduction in the muscle fiber diameter, varying sizes of fibers, and fibers with centronuclei and inflammatory cell infiltration (Figure 3, white arrow). However, these muscle atrophic features were attenuated in RJ-treated mice (Figure 3). The frequency peak of the fiber cross-sectional area (CSA) shifted from 1800 to 1200 µm^2^ with age (Figure 3i). However, the fibers remained large (more than 2100 µm^2^) in RJ-treated mice (Figure 3i). The mean fiber CSA was also reduced in aged control mice but was not significantly different from that in RJ-treated mice (Figure 3j). Furthermore, the significant increase in the prevalence of centronuclear myofibers with age tended to decrease in RJ-treated aged mice (Figure 3k). These results show that RJ slightly affected the age-related morphological changes in muscle fibers.

### 3.4. Effects of RJ on the Age-Related Changes in the mRNA Expression of Muscle Regeneration and Degradation-Related Genes 

**Figure 4:**
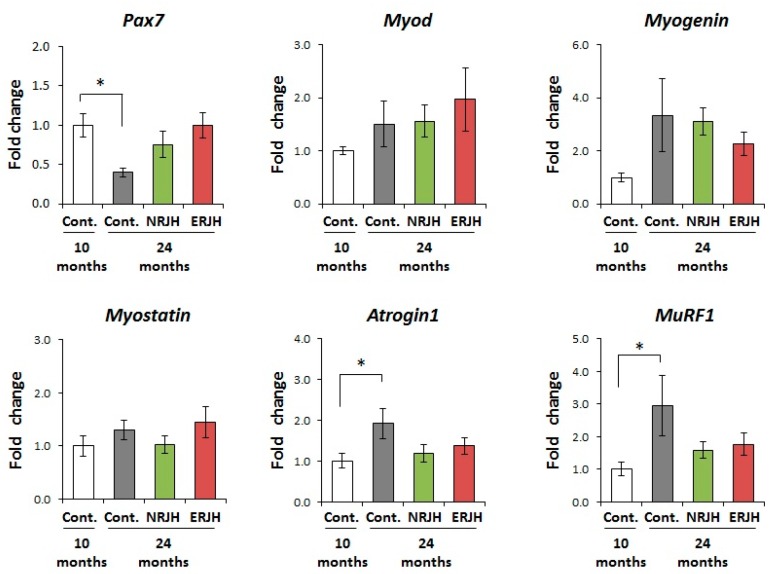
Effects of RJ on the mRNA expression of muscle differentiation, regeneration, and atrophy-related genes in genetically heterogeneous mice. The mRNA expressions of pax7, Myo D, Myogenin, Myostatin, Atrogin1, and MuRF1 in the gastrocnemius muscle were measured by real-time reverse transcription polymerase chain reaction. The data were normalized with respect to a house-keeping gene and shown as the mean fold-change ± SEM (*n* = 5) with respect to the control group at 10 months of age (Control, 10 months). *P* values were determined by the Kruskal–Wallis test (post-hoc Dunn’s multiple comparisons test). * *P* < 0.05.

To further investigate the mechanisms underlying the improvement in the motor function by RJ, we analyzed the effects of RJ on the expression of genes regulating muscle mass and functions. We first examined the effect of RJ on the satellite cell marker Pax7. Satellite cells are muscle stem cells and play important roles in muscle regeneration. The number of PAX7-positive satellite cells is reported to decrease with age [30]. In this study, the expression of Pax7 significantly decreased with age in control mice. However, the expression of Pax7 in aged RJ-treated mice was comparable to that in young mice (Figure 4). We next examined the effects of RJ on the muscle differentiation genes MyoD and myogenein [31] and the muscle growth regulator myostatin [32]. There were no significant differences in the expression of MyoD, myogenine, and myostatin among the groups (Figure 4). We also examined the effects of RJ on the expression of the muscle-specific E3 ubiquitin ligases MuRF1 and atrogin-1 (also known as MAFbx), which are reported to be upregulated in various muscle atrophy models, including models of aging [33]. We confirmed that both MuRF1 and atrogin-1 significantly increased with age in HET mice. However, in RJ-treated mice, the expressions of MuRF1 and atrogin-1 were similar to those in young mice (Figure 4). These results suggest that RJ delayed the age-dependent deregulation of muscle quality by regulating both muscle regeneration and catabolic genes.

## 4. Discussion

In this study, we investigated the effects of a lifelong treatment with RJ on the lifespan and healthy lifespan during normal aging using HET mice. First, we investigated the effect of RJ on the lifespan expectancy. The median lifespan of control HET mice in our study was 25.7 months (782 days). The range of the median lifespan of male HET mice in previous studies was 739 to 876 days [16,34,35], suggesting that the animals in our study were comparable to those in previous studies. In addition, we also observed a wide range of pathologies at necropsy, including both neoplastic and non-neoplastic diseases. These observations confirmed the genetic heterogeneity of this mouse model. In a previous study using C3H/HeJ male mice, RJ supplementation (60 mg/kg body weight/day) successfully prolonged the median lifespan by 23.6% compared with control animals [19]. As in Inoue’s study, we treated HET mice with RJ at a concentration of 0.05% (approximately 50 mg/kg body weight/day) and 0.5% (approximately 500 mg/kg body weight/day) from 6 months of age. We also compared the longevity effect of NRJ with ERJ, which was developed to avoid allergic reactions associated with NRJ. We observed that neither NRJ nor ERJ could prolong the lifespan, regardless of the RJ dose. Our experiments differ in some respects from others, regarding the mouse strain used, the number of mice, the starting time of RJ treatment, and the breeding conditions. In addition, neither the present nor previous studies used female mice. Further experiments are required to clarify the role of RJ in extending the lifespan.

Sarcopenia is defined as a decrease in the physical performance and skeletal muscle mass with age. In aging societies, sarcopenia is directly associated with frailty, which leads to a reduced QOL. In our HET model, we observed features of sarcopenia from 30 months of age, such as impairment of motor coordination (Figure 2) and loss of muscle strength and weight (Figure 2 and Supplementary Figures S3 and S5). In this study, the dietary supplementation with a high dose of RJ (both NRJ and ERJ) significantly improved the motor functions in the course of normal aging (Figure 2) without changing the muscle weight. We did not observe any differences in the motor functions between control and low-dose-treated RJ groups at 36–39 months of age (Supplementary Figure S2). We also noted that the number of survived mice was not high enough for further statistical analysis (NRJL, *n* = 1; ERJL, *n* = 3). On the basis of findings of both in vitro and in vivo studies, food components are expected to prevent the progression of sarcopenia [36], but the effects of these food components on sarcopenia in normal aging have not been elucidated. In NIA’s ITP, the effects of food components such as curcumin, green tea extract, resveratrol, medium-chain triglycerides, and fish oil on the lifespan have been tested during normal aging; however, these food ingredients failed to extend the lifespan or healthy lifespan and to improve the motor functions [26,27,34]. In this study, muscle fiber atrophy was partially affected by a lifelong treatment with RJ, although the muscle weight was not restored. Previous reports have shown that a short-term treatment with 5% RJ in aged C57BL/6 mice prevented the progression of sarcopenia, including the loss of both muscle weight and strength [21]. Although the dose of RJ was 10 times lower in our study than in Niu’s study, a lifelong treatment with RJ certainly affected the motor functions. We have not fully clarified the mechanism of the prevention of motor function impairment with aging by administration with RJ. Structural disruptions in neuromuscular junctions (NMJ) are thought to be related to motor function impairment during aging, because the disruption of NMJ leads to synaptic dysfunctions in muscles. It is reported that the expression of NMJ-related genes that regulate nerve impulse transmission is altered with aging [37]. However, in our preliminary data, the expression of NMJ-related genes did not change with aging and also was not affected by RJ administration (data not shown). It is reported that RJ contains acetylcholine [38]. Recently, RJ was shown to induce vasorelaxation through an acetylcholine-dependent mechanism in vivo [39]. These results suggested that RJ directly affect NMJ in an acetylcholine-dependent manner. Further studies, such as the histological analysis of NMJ after RJ treatment, are required to clarify the mechanism of action of RJ in motor function improvement. Recent human clinical trials in elderly people have shown that RJ has the potential to attenuate the decline in the grip strength occurring with age [40]. These results indicate that RJ is a food supplement that is expected to prevent sarcopenia in aging individuals.

Age-related muscle atrophy is triggered by the disruption of the balance between muscle regeneration and muscle degradation [41]. Muscle-resident stem cells, known as satellite cells, support muscle regeneration [42]. Satellite cells are thought to be associated with the progression of muscle atrophy, as the number of satellite cells is reduced in the aging process [30]. Furthermore, recent depletion studies have shown that satellite cells also contribute to age-related NMJ degeneration [43]. Therefore, the maintenance of satellite cell stemness is thought to be a strategy for preventing muscle function decline [36]. Since it is known that satellite cells are heterogeneous, several satellite cells markers, such as Pax7 and Pax3, are used to identify them [41]. In a study describing FACS-isolated single satellite cells, all muscle stem cells expressed Pax7. However, 10% of the investigated satellite cells expressed Pax3 [44]. These results suggested that Pax7 is the canonical marker for satellite cells. In the present study, we showed that the age-related reduction in Pax7 expression was attenuated by treatment with RJ. Niu et al. also reported that RJ increased satellite cell growth and differentiation via IGF-1–Atk signaling in vitro. In addition, treatment with RJ increased the number of satellite cells in aged mice [21]. These results suggest that the maintenance of the satellite cell function using RJ helps prevent the decline in the motor functions associated with sarcopenia. The disturbance of the muscle ubiquitin proteasome system is thought to be another cause of the development of muscle atrophy with age. There are two major muscle-specific E3 ubiquitin ligases: MuRF1 and MAFbx/atrogin-1. It is reported that both MuRF1 and MAFbx/atrogin-1 are upregulated in response to muscle atrophy-inducing statuses, including aging [33]. In our study, there are large variations in gene expression among animals because we used genetically heterogeneous aged mice. In such experimental conditions, we found that both MuRF1 and atrogin-1 were transcriptionally increased with age in HET mice, and RJ treatment affected this age-related upregulation (Figure 4). The expression of MuRF1 and atrogin-1 is reportedly regulated by various stressors, including age-related signaling such as oxidative stress and inflammation [33,45,46]. RJ is known to exert anti-oxidant effects in various stress models [47,48,49]; indeed, the RJ-specific fatty acids (E)-10-hydroxy-2-decenoic acid (10H2DA) and 10-hydroxydecanoic acid (10HDA) have anti-inflammatory functions [50]. To understand the effects of RJ on catabolic genes, we have planned in vitro studies. The related results will be reported in a separate manuscript. 

## 5. Conclusions

In the present study, we showed that RJ attenuated the progression of age-related motor function decline using HET mice. RJ affected the histological and molecular changes normally occurring with age in muscles, suggesting that RJ preferentially affected muscle quality over muscle quantity. These results show that RJ may be useful for improving the QOL through the attenuation of age-related motor function impairment.

## Supplementary Materials

The following are available online at http://www.mdpi.com/2072-6643/10/9/1191/s1, Figure S1: Changes in food intake and body weight with aging in male HET mice, Figure S2: Changes in the motor functions during aging in HET mice, Figure S3: Changes in the muscle weight and bone mineral density during aging in male HET mice, Figure S4: Generation of genetically heterogeneous (HET) mice, Table S1: General composition of the experimental diets, Table S2: Effects of RJ on the plasma biochemical parameters, Table S3: Effects of RJ on the number of blood cells, Table S4: Sequences of primers used in this study.
