# Supplementary material for: Royal Jelly Delays Motor Functional Impairment During Aging in Genetically Heterogeneous Male Mice
Source: Nutrients. 2018 Sep 1;10(9):1191. doi: 10.3390/nu10091191 (PMC6164577; doi:10.3390/nu10091191)
Supplement: Supplementary file 1 [file nutrients-10-01191-s001.pdf]

nutrients

Royal Jelly Delays Motor Functional Impairment During Aging in Genetically Heterogeneous Male Mice

Authors:

Nobuaki Okumura[1], Toshihiko Toda[2], Yusuke Ozawa[2], Kenji Watanabe[2], Tomoki Ikuta[1,] Tomoki Tatefuji[1], Ken Hashimoto[1], Takahiko Shimizu[2]

Table S1. General composition of the experimental diets.

Diets	Control (Cont.)	0.05% NRJ (NRJL)	0.5% NRJ (NRJH)	0.05% ERJ (ERJL)	0.5% ERJ (ERJH)
Moisture (%)	7.9	7.9	7.9	7.9	7.9
Protein (%)	23.1	23.1	23.1	23.1	23.1
Lipid (%)	5.1	5.1	5.1	5.1	5.1
Ash (%)	5.8	5.8	5.8	5.8	5.8
Carbohydrate (%)	58.1	58.1	58.1	58.1	58.1
Energy (kcal)	359.0	359.0	359.2	359.0	359.1

Table S2. Effects of RJ on plasma biochemical parameters.

Parameter	10 months	24 months		
	**Control**	**Control**	**NRJH**	**ERJH**
Albumin (g/dL)	2.9 ± 0.23	2.9 ± 0.79	2.6 ± 0.11	2.5 ± 0.17
AST (U/L)	74.4 ± 27.7	102.8 ± 63.6	81.6 ± 27.6	71.8 ± 15.2
ALT (U/L)	27.8 ± 4.3	49.6 ± 49.7	25.0 ± 9.3	19.6 ± 2.9
T-CHO (mg/dL)	135 ± 27.7	134 ± 126.3	102 ± 33.7	68 ±12.9 *
TG (mg/dL)	71 ± 30.6	38 ± 11.4	32 ± 8.7	30 ± 12.2 *
Glucose (mg/dL)	78.2 ± 6.5	75.4 ± 7.7	73.2 ± 5.7	86.9 ± 7.1

Mean ± SEM. * $P < 0.05$ vs 10 months, control

Table S3. Effects of RJ on the number of blood cells.

Parameter		10 months	24 months				
		**Control**	**Control**	**NRJL**	**NRJH**	**ERJL**	**ERJH**
WBC	$\times 10^2$ /ml	11.4 ± 6.2	57.8 ± 36.3	16.5 ± 11.2	8.2 ± 4.7	9.4 ± 6.1	5.4 ± 3.7
RBC	$\times 10^4$ /ml	977 ± 36	839 ± 55	930 ± 150	872 ± 55	869 50	797 ± 30
HGB	g/dL	12.6 ± 0.5	10.3 ± 0.6	11.4 ± 1.9	10.8 ± 0.6	11.0 ± 0.5	10.3 ± 0.5
HCT	%	47.6 ± 2.0	38.8 ± 2.3	43.0 ± 6.6	41.0 ± 2.1	41.1 ± 2.1	37.8 ± 1.5
MCV	fL	48.8 ± 1.3	46.5 ± 1.2	46.5 ± 0.7	47.2 ± 0.6	47.4 ± 0.5	47.4 ± 0.8
MCH	pg	12.9 ± 0.4	12.4 ± 0.3	12.2 ± 0.2	12.5 ± 0.2	12.7 ± 0.2	12.9 ± 0.3
MCHC	g/dL	26.5 ± 0.3	26.7 ± 0.3	26.2 ± 0.4	26.3 ± 0.3	26.8 ± 0.5	27.2 ± 0.6
PLT	$\times 10^4$ /ml	121 ± 7.2	132 ± 22.3	122 ± 18.6	144 ± 6.8	132 ± 3.3	140 ± 8.9

Mean ± SEM

Table S4. Sequences of primers used in this study.

Gene	Symbol	Accession No.		Sequence (5' > 3')	Product length
Tripartite motif-containing 63	MuRF1	NM_001039048	F	gccatcctggacgagaagaa	234
			R	cagctggcagcccttgga	
F-box protein 32	Atrogin-1	NM_026346	F	agaccggctactgtggaagag	217
			R	ccgtgcatggatggtcagtg	
Myogenic differentiation 1	Myod	NM_010866	F	ggctacgacaccgcctacta	110
			R	gtggagatgcgctccactat	
Paired box 7	Pax7	NM_011039	F	gacgacgaggaaggagacaa	110
			R	acatctgagccctcatccag	
Myogenin	Myog	NM_031189	F	ccttgctcagctccctca	94
			R	tgggagttgcattcactgg	
Myostatin	Mstn	NM_010834	F	ctgtaaccttcccaggacca	197
			R	tcttttgggtgcgataatcc	
Beta-2 microglobulin	B2m	NM_009735	F	ctggctcacactgaattcaccc	104
			R	atgtctcgatcccagtagacgg	
Actin, beta	Actb	NM_007393	F	ttcttgggtatggaatcctgtggc	85
			R	agaggtctttacggatgtcaacg	
histone cluster 2, H2aa1	H2a	NM_013549	F	acgaggagctcaacaagctg	105
			R	tatggtggctctccgtcttc	
Glucuronidase, beta	Gurb	NM_010368	F	tgaacgggaagcaatcctgc	133
			R	acctccctcatgttccaccac	
Ribosomal protein S18	Rsp18	NM_011296	F	gatgggcggcggaaaatagc	107
			R	gccctcttggtgaggtcgat	

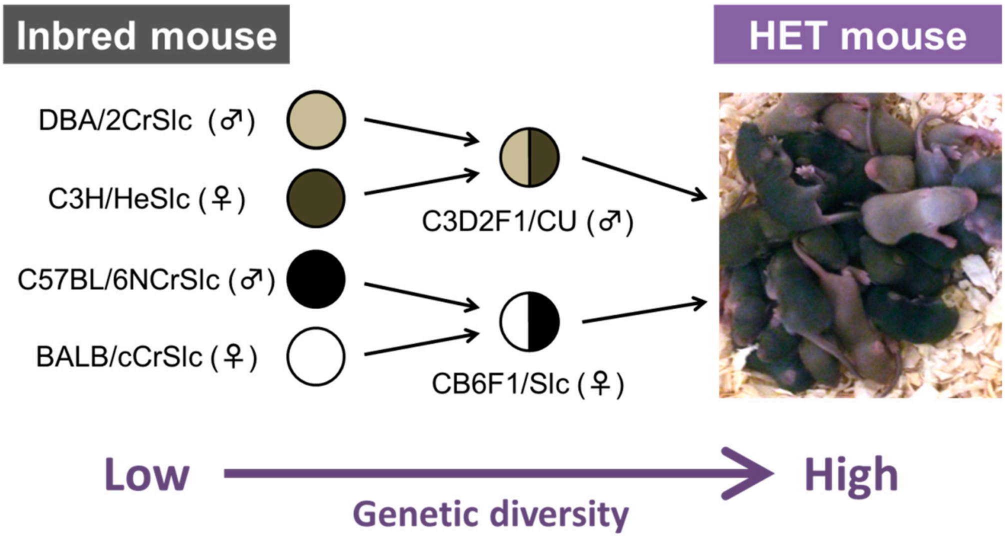

Figure S1. Generation of genetically heterogeneous (HET) mice.

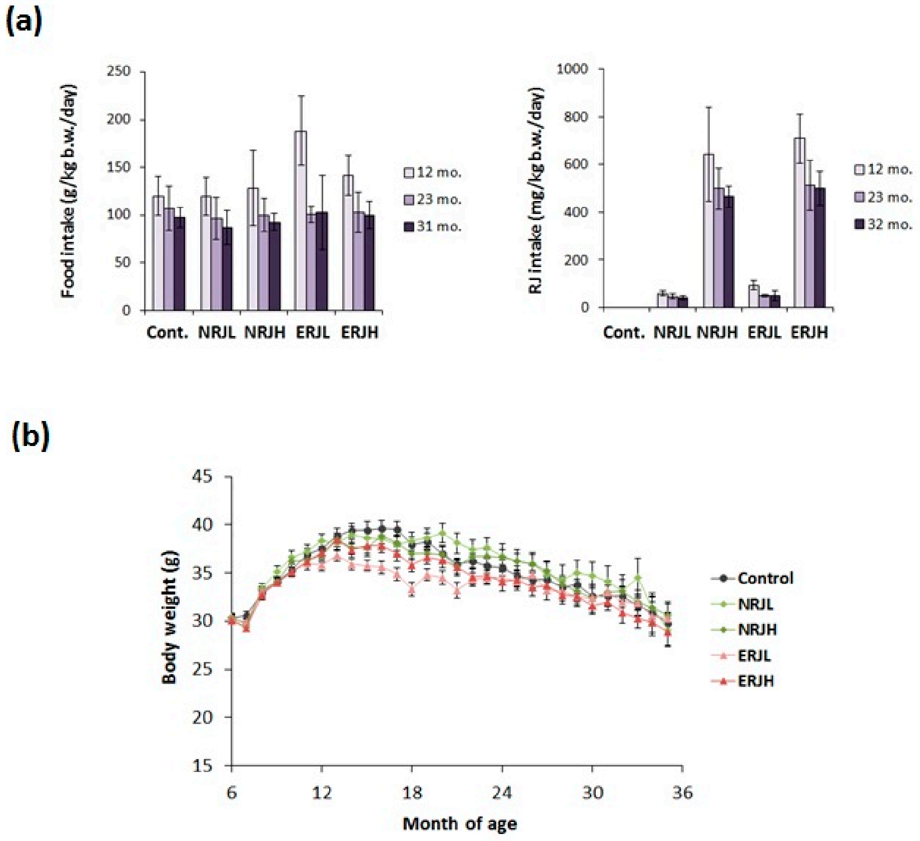

Figure S2. Changes in food intake and body weight with aging in genetically heterogeneous male mice. (a) Changes in the food intake and RJ intake during the experiments. The food intake (left) was measured at 12, 23, and 31 months of age. The daily RJ intake was calculated from the food consumption. The data are shown as the mean ±SD (n = 5). (b), The effects of royal jelly on the body weight. The body weight of mice alive at each time point was measured every month. The data are shown as the mean ± SD.

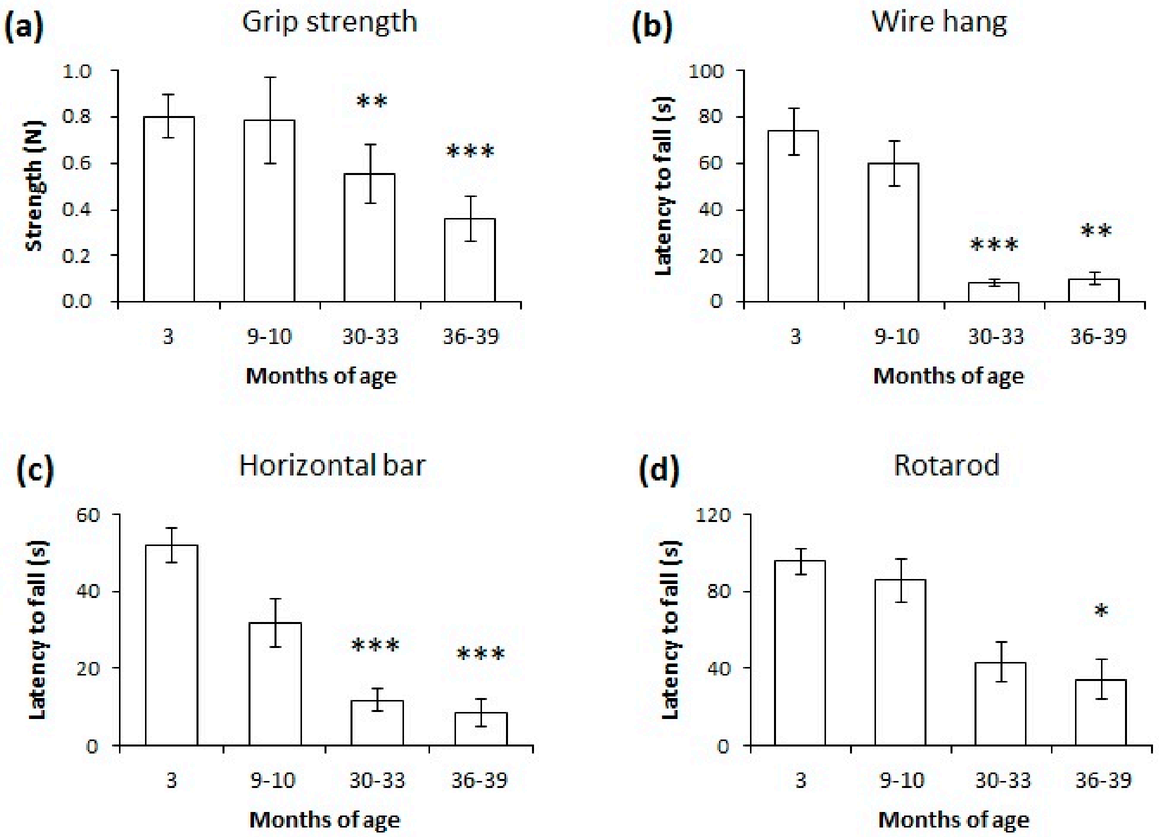

Figure S3. Changes in the motor functions during aging in HET mice. Grip strength was measured by a spring gauge test (a) and a wire hang test (b). Locomotor activity was measured by a horizontal bar test (c) and an accelerated rotarod test (d). Data are shown as the mean ± SEM. ** $P < 0.01$ and * $P < 0.05$ vs. 3 months.

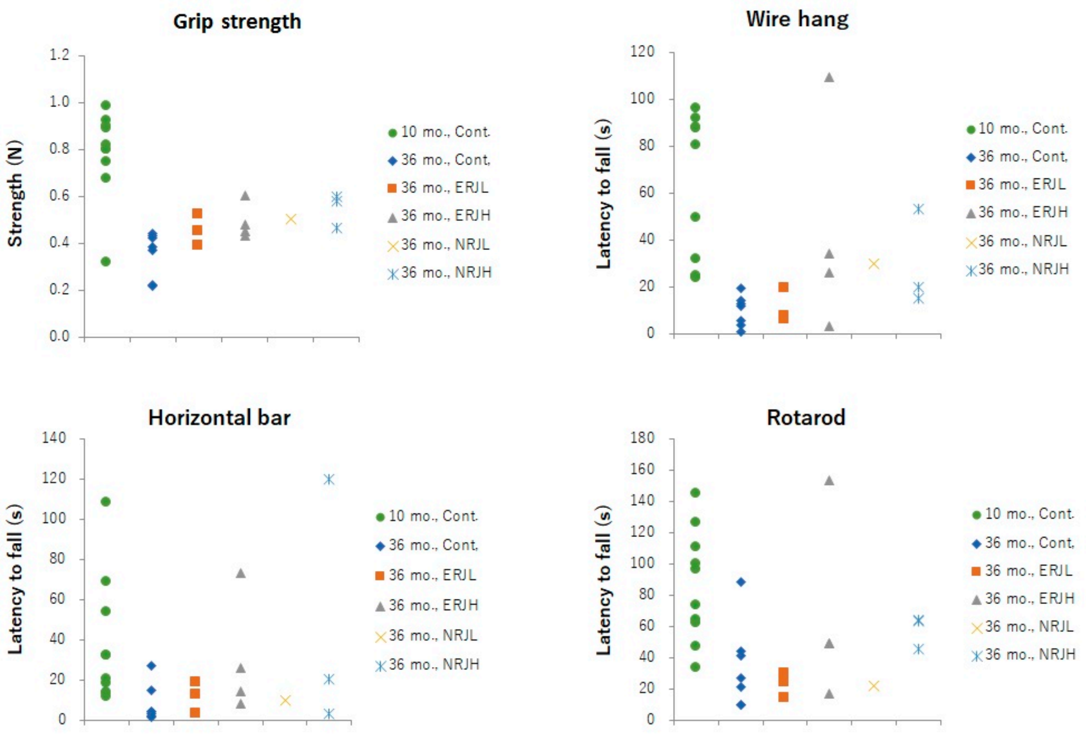

Figure S4. Effect of royal jelly on the motor functions at 36–39 months of age in HET mice. The motor functions of mice at 36–39 months of age (indicated as 36 mo. in the figure) were measured by different physical performance tests. The grip strength was measured by a spring gauge test (Grip strength) and a wire hang test (Wire hang). The locomotor activity was measured by a horizontal bar test (Horizontal bar) and an accelerated rotarod test (Rotarod). Each plot shows the results from individual mice.

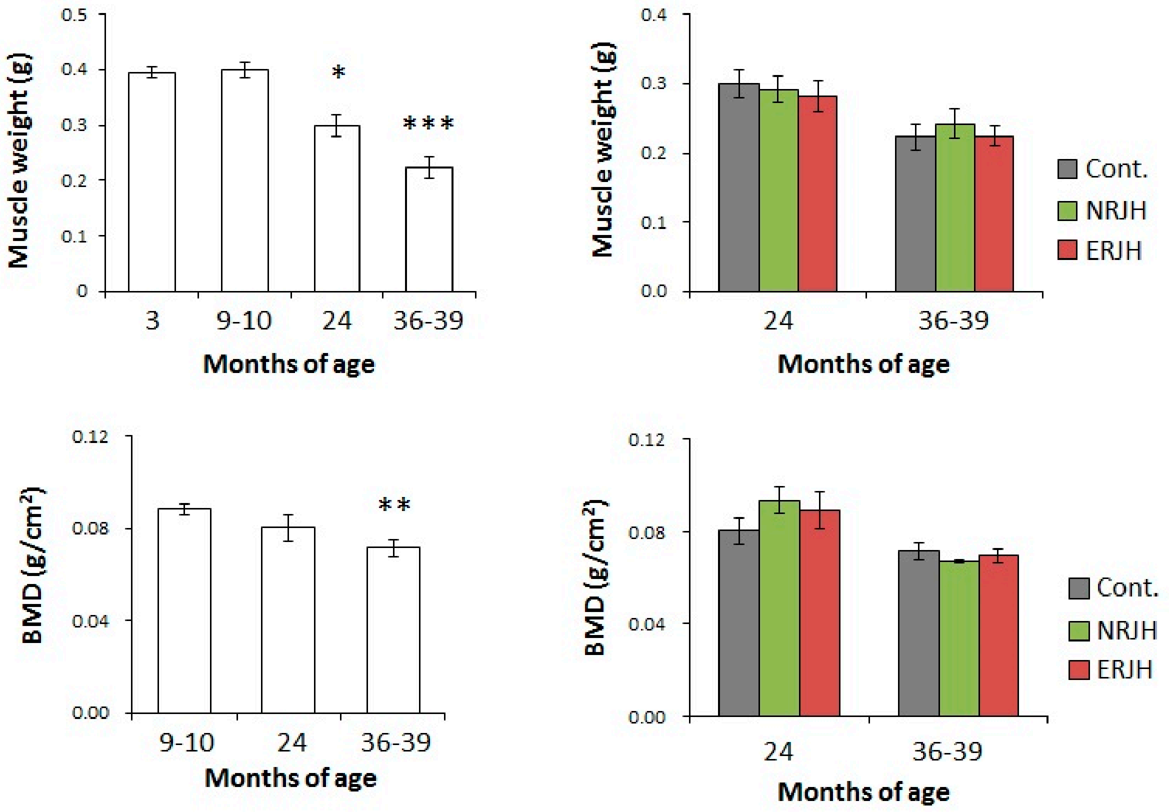

Figure S5. Changes in the muscle weight and bone mineral density during aging in HET mice. The upper panels show the muscle weight at each time point. Data are shown as the mean ± SEM. *** $P < 0.001$, ** $P < 0.01$ and * $P < 0.05$ vs. 3 months. The lower panels show the bone mineral density (BMD). Data are shown as the mean ± SEM. *** $P < 0.001$, ** $P < 0.01$ and * $P < 0.05$ vs. 9-10 months.
